# Concordance of vestibular test batteries in patients with vestibular neuritis

**DOI:** 10.55730/1300-0144.5504

**Published:** 2022-07-09

**Authors:** Burak KABİŞ, Hakan TUTAR, Emre GÜRSES, Bülent GÜNDÜZ, Songül AKSOY

**Affiliations:** 1 Department of Audiology, Faculty of Health Science, Gazi University, Ankara, Turkey; 2 Department of Otolaryngology Head and Neck Surgery, Faculty of Medicine, Gazi University, Ankara, Turkey; 3 Department of Audiology, Faculty of Health Science, Hacettepe University, Ankara, Turkey

**Keywords:** Vestibular neuritis, vestibulo ocular reflex, superior vestibular nerve, inferior vestibular nerve, kappa analysis

## Abstract

**Background/aim:**

A growing number of vestibular function tests are utilized to differentiate and verify the diagnosis of vestibular neuritis. The aim of this study is to retrospectively investigate the consistency of the results of the objective vestibular test batteries in patients with a preliminary diagnosis of vestibular neuritis.

**Materials and methods:**

We reviewed a total of 37 adult patients (mean age: 39.03 ± 11.67, 19 females, 18 males) who met the inclusion criteria with a prediagnosis of vestibular neuritis from 379 patients suffering vestibular symptoms. Caloric test (CVT), video head impulse test (vHIT), and ocular and cervical VEMP tests were compared with Cohen’s kappa (Κ) analysis according to the likely affected part of the vestibular nerve.

**Results:**

The highest statistically significant K value was found between horizontal vHIT and ocular VEMP (K = 0.707; good grade, *p* < 0.05). All the tests compared with CVT were poorly in agreement (K = 0.288; 0.262; 0.256 for HvHIT, oVEMP, AvHIT, respectively, *p <* 0.05).

**Conclusion:**

VEMP and vHIT tests have prominent diagnostic value and agree with each other for detecting and differentiating the types of vestibular neuritis. Further studies should aim to include cutting-edge technologies such as functional HIT and ocular counter roll test.

## 1. Introduction

There has been a growing demand for vestibular function evaluation test batteries in the clinical assessment of peripheral vestibular integrity in multiple disorders, including vestibular neuritis (VN), acoustic neuroma, superior semicircular canal dehiscence syndrome, and Ménière’s disease (MD). Although the diagnosis is made based on clinical features, vestibular function tests are fundamental to differentiate and verify the diagnosis. The most widely accepted evaluating tests of vestibular function are video head impulse test (horizontal canal [HvHIT], posterior canal [PvHIT], anterior canal [AvHIT]), vestibular-evoked myogenic potentials (ocular [oVEMP], cervical [cVEMP]), and videonystagmography including caloric vestibular test (CVT) [1].

The vast majority of test batteries aim to assess the same anatomical localization. However, in some cases, the results of the tests differ. At the same time, compatible results have been found in others. Although the dissimilarities are explained by frequency domain difference [2], few studies have addressed the concordance of these tests, including oVEMP and cVEMP.

In a study conducted with 172 patients having more than 25% canal paresis and suffering balance disorder to understand the sensitivity of test batteries, CVT and vHIT test results were compared with the results of studies reporting a vHIT sensitivity of 41% and specificity of 92% [3]. They conclude that in patients with severe canal paresis, the vHIT is insensitive to vestibular discrepancies. Similarly, in another study conducted with 60 participants with dizziness, it was reported that the agreement between canal paresis and gain asymmetry was low, and the correlation coefficient between them was 0.67 (K= 0.252) [4]. However, it is an important limitation in both studies that the participants did not have a definitive diagnosis, and the tests were performed in individuals with different types of vestibular disorders. To better understand the relationship between test batteries, it would be better if the patient groups were homogeneous.

In studies with a certain diagnosis, it is found that CVT and vHIT disassociation are instrumental hallmarks of MD [5] and are also found in patients with the recovery phase of VN [6]. So far, the published comparative studies have mainly focused on vHIT and CVT in patients with and without definite diagnoses. The overall agreement is that there is a poor correlation between these two tests, and both should be used as complementary [7]. However, to understand the comprehensive relation of VOR in patients with vertigo, VEMP tests are also useful and should be involved in statistical analyses [8]. Therefore, in the current study, we aimed to investigate the sensitive factors used in vestibular test batteries to diagnose inferior and superior vestibular neuritis. For this purpose, CVT, vHIT, oVEMP, and cVEMP association were evaluated in patients with vestibular neuritis. The findings were grouped according to the locations where the vestibular nerve was evaluated. The CVT, oVEMP, HvHIT, and AvHIT were used for the evaluation of the superior branch of the vestibular nerve, and cVEMP and PvHIT were used for the evaluation of the inferior branch of the vestibular nerve.

## 2. Materials and methods

### 2.1. Participants

**Figure 1:**
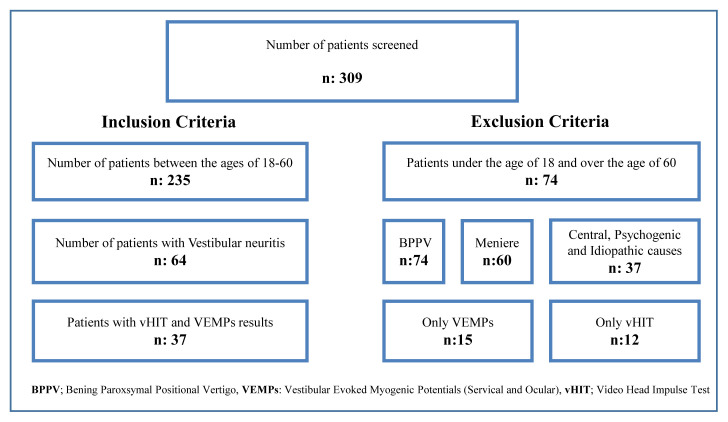
Flow diagram showing the given scan results to be used for the study.

This study includes a retrospective analysis of 309 patients with vertigo who applied to University Faculty of Medicine Department of Otorhinolaryngology and Audiology within 1 year and underwent comprehensive vestibular and audiological evaluations. The procedure was approved by the Clinical Research Ethics Committee at Gazi University (decision no. 788). Patients diagnosed with vestibular neuritis between 18 and 60 years old were included in the study. Patients diagnosed with any other identifiable vestibular disorders, taking medication with the side effects of vertigo, incomplete medical records, having cervical spine injury, or having limited movement of the neck were excluded. Although the initial population was 309, a statistical evaluation was carried out with 37 patients (Figure 1).

A total of 37 patients (19 female, 18 male, aged 18–60 years [mean: 39.03 ± 11.67]) were retrospectively included in the study. To account for age, we classified patients into three groups. The patients’ demographic information is shown in Table 1. The diagnostic criteria and types of VN were determined based on the study proposed by Magliulo et al. [9]. Since there are interexaminer differences of the VOR gain values for the vHIT, an experienced audiologist performed all tests [10].

### 2.2. Videonistagmography

A videonystagmography (VNG) test battery was used to rule out central and peripheral pathologies that might be observed in addition to VN. First, each patient was seated on a fixed chair 120 cm from the test screen, and a calibration was performed. Since VNG comprises a series of subtests, the order of application of the subtests were as follows: spontaneous nystagmus, gaze nystagmus, saccade, smooth pursuit, optokinetic nystagmus tests, head-shaking nystagmus tests, positional and positioning tests, and CVT.

### 2.3. Caloric vestibular test

Bithermal (50 °C and 27 °C) air caloric vestibular test (CVT) was applied with reference to British Society of Audiology procedures [11]. During the test, the patients were positioned in the supine position on the stretcher with the head at 30° of flexion. The maximum velocities of the slow phases of the nystagmus observed in the findings were calculated using the Jongkees’ formula at bilateral and both temperatures. Canal paresis value was determined for each patient. Results of 25% and above were accepted as canal paresis.

### 2.4. Video head impulse test

Video head impulse test (vHIT) measurements were conducted with an experienced audiologist. The test phase consists of three sections as Horizontal (Left-Right) for the evaluation of the horizontal canals, RALP (right anterior-left posterior) and LARP (left anterior-right posterior) for the evaluation of the vertical canals. Results were grouped as horizontal vHIT (HvHIT), anterior vHIT (AvHIT), posterior vHIT (PvHIT).

During these tests, passive pushing force was applied to the patient’s head at angles of approximately 15°–20° relative to the tested semicircular canal (SCC). During the test, the volunteer was asked to free his head and fix his eye at the point set on the wall at a distance of 1.2 m during the head movements.

During the HvHIT evaluation phase, the participant’s head was flexed to 30 degrees, and during head movement, he was asked to look fixedly at the target opposite him. Head movements were applied with 15-degree angles in the yaw plane.

In the evaluation of vertical SSK (AvHIT and PvHIT), the participant’s head was turned 45° to the right or left during the test, the optimum stimulation position of the vertical channels was adjusted, and the thrust force was applied. During the evaluation of the horizontal and vertical canals, ten head thrusts were applied for each canal.

The VOR gain value of each canal and the asymmetry value between channel pairs were analyzed. The manufacturer’s test protocol and normative values were taken as references. VOR gain values of 0.76 and below was considered abnormal, and for the asymmetry between canal pairs, 8% and above were considered abnormal values [12].

### 2.5. Vestibular evoked myogenic potentials

Ocular VEMP (oVEMP) and cervical VEMP (cVEMP) tests were performed, two hundred fifty click stimuli were presented at 90 dB nHL with insert earphones (127 db SPL, duration: 0.1 ms) for both evaluations. In the cVEMP test, patients were asked to sit on a fixed chair, turn their head towards the contralateral shoulder, and keep the muscle contraction constant throughout the test. Therefore, they fit the electromyographic activation criteria (50–200 μV).

In the oVEMP test, the patients were asked to look upward while in the sitting position, aiming at the predetermined object that forms an angle of 30° with the eyes on the horizontal axis, and the contralateral eye responses were recorded. The threshold was determined so that the same waveform and latency were obtained in a minimum of two consecutive tests. The peaks of the first waveform formed after stimulus administration were determined as n1 and p1. Peak latency, peak-to-peak amplitude, and asymmetry ratio (AR) values of the waves were calculated. ARs above 30% or below −30% and above 40% or below −40% were accepted abnormal for cVEMP and oVEMP, respectively [13].

### 2.6. Statistical analysis

The data were analyzed using a statistical program. To determine whether the variables were normally distributed, visual (histograms, probability plots) and analytical methods (Kolmogorov-Smirnov) were performed. Continuous variables were presented as the mean ± SD (normally distributed). Whether the test battery results differed according to sex was investigated by t-test in independent groups. Cohen’s kappa coefficient value was calculated to examine the compatibility of the test results with each other. Kappa result is interpreted as follows: values ≤ 0 as indicating no agreement; 0.01–0.20 as none to slight; 0.21–0.40 as fair; 0.41–0.60 as moderate; 0.61–0.80 as substantial; and 0.81–1.00 as almost perfect agreement. The significance level was recognized as *p* < 0.05.

## 3. Results

We detected no significant between age and sex differences for any of the test parameters (*p* > 0.05).

In the oVEMP test, abnormal findings were observed in 20 of the patients, while normal findings were observed in 17 patients. For the right and left ears, normal findings were found in 28 and 26 patients (R: 75.7%, L: 70.3%), and pathological findings in 9 and 11 patients (R: 24.3%, L: 29.70%), respectively. In the cVEMP test, normal findings were obtained for the right and left ears in 21 patients each, while pathological findings were obtained in 16 patients for each ear (R: 43.24%, L: 43.24%).

**Figure 2:**
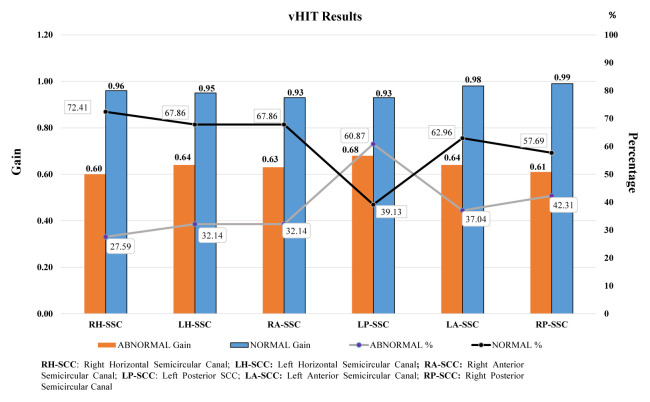
Bar chart showing vHIT findings.

vHIT results showed that the rate of posterior SCC abnormal findings (LP-SCC: 37.80%; RP-SCC: 29.70%) was higher than other SCCs. The gain values of all SCCs were between 0.60 and 0.68 in patients with abnormal vHIT. The average percentage and gain values of normal and abnormal values of all SCCs are shown in Figure 2.

**Figure 3:**
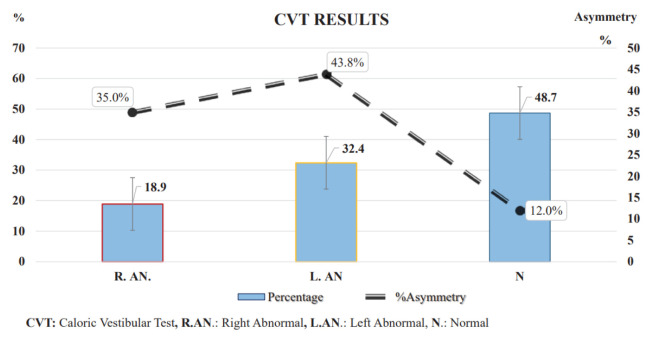
Bar chart showing CVT findings.

According to the VNG results, oculomotor tests were normal in all participants. In the positional tests, Dix-Hallpike and Roll were also negative in all patients. In CVT, 18 patients (48.6%) had normal findings (canal paresis <25%: mean: 12 ± 8.03%), and 19 patients (51.4%) had abnormal findings (canal paresis > 25%). While seven (18.9%) of the patients with abnormal CVT results had right asymmetry (canal paresis: 35 ± 14.15%), 12 (32.4%) had left asymmetry (canal paresis: 43.83 ± 9.79%). The CVT results of the patients are shown in Figure 3.

While HvHIT, AvHIT, oVEMP, and CVT findings were analyzed to evaluate the superior branch of the vestibular nerve, PvHIT and cVEMP findings were analyzed to evaluate the inferior branch. The applied tests were compared in pairs according to the regions of the vestibular nerve they evaluated. The degrees of agreement between them were calculated using Cohen’s kappa coefficient value. Accordingly, while the highest agreement (*good degree*) among the tests was found between the oVEMP and HvHIT tests evaluating the superior vestibular nerve (K: 0.707, *p* < 0.05), the lowest agreement (poor degree) was found between the CVT and AvHIT (K: 0.256, *p* < 0.05). When the agreement of the tests evaluating the superior vestibular nerve was analyzed, the tests compared with the CVT were a generally poor degree. In contrast, a moderate agreement (K: 0.594, *p* < 0.05) was found between cVEMP and PvHIT when evaluating the inferior vestibular branch. Moreover, when the agreement between the tests was examined in terms of evaluating the whole vestibular nerve, the agreement between the VEMP tests and the vHIT tests was almost the same and at a good level (K: 0.612 and 0.682, respectively). Table 2 shows the agreement between the tests and the common observed abnormal and normal percentage values.

## 4. Discussion

Vestibular neuritis can affect the superior and inferior vestibular nerves selectively or affect both. Therefore, all objective test batteries are needed to distinguish types of VN or reveal diagnostic hallmarks of VN. In the current study, 19 (51.35%) participants had a caloric weakness, whereas 17 participants (45.94%) had an abnormal hvHIT. It can be concluded that the CVT is slightly more sensitive than vHIT. The findings correspond with other peripheric vestibular disorders, such as Meniere’s disease (caloric weakness: 44.6%, abnormal vHIT: 25.9%) and sporadic unilateral vestibular schwannoma (caloric weakness: 72%, abnormal vHIT 44%) [14]. However, although CVT was more sensitive, the numbers of pathological vHIT and CVT results were obtained closer to each other in our study. This may be because the tests were performed on the participants within the first 10 days after the onset of symptoms. Bartolomeo et al. found that there is normalization in the results of vHIT in the chronic period [6]. The differences among studies can be explained by the different duration of applied vestibular tests. Also, since some of our participants had inferior canal neuritis, it can be expected that CVT results would be found more sensitive in the study group that consisted of only superior vestibular neuritis.

The main aim of the current study was to identify the best comparative agreements among the vestibular test batteries in patients with vestibular neuritis. We found that there was a poor agreement between HvHIT, AvHIT, and CVT. These results could easily be explained by the different frequency domains provided by these systems. The primary physiological function is to initially stabilize the gaze and during head movement at a high frequency of stimulation which is evaluated by vHIT. Whereas at low frequencies of stimulation, velocity storage, smooth pursuit, and optokinetic nystagmus provide help to the VOR, which is evaluated by CVT [4, 15]. Although the same disassociation was found in a study performed in 51 patients with Meniere disease as our study, the authors suggested that the vHIT test should be used as a screening tool and the CVT is only considered when vHIT results are normal [16]. A similar conclusion was suggested by another study: that a vHIT might be performed first and, if unremarkable, a caloric examination should then be undertaken [3]. In a study with 29 patients with VN, it was found that using vHIT as a screening tool has reduced the need for caloric testing in 51% of cases [6]. This is understandable since CVT is time-consuming and stressful for patients. However, because of the weak kappa coefficient values reported here, these tests should not be used interchangeably. Also, it is well known that regular hair cells transmitting more information from low-frequency head movement and irregular hair cells transmitting the information from high-frequency head movements are both affected in VN. Since the level of impairment by inflammation is not known for the different types of hair cells, both tests are needed.

Four conditions have been identified for the type of VN: entire (superior and inferior division), vestibular neuritis (EVN), superior vestibular neuritis (SVN), inferior vestibular neuritis (IVN), and ampullary vestibular neuritis (AVN) [17]. Although it is similar to this study, our study aimed to examine the compatibility of different test methods in terms of parts of the vestibular nerve. In our study, the evaluated parts of the vestibular nerve (whole, inferior, and superior vestibular nerve) were classified into three different groups as sVN, iVN, and wVN (seen in Table 2). When the test pairs providing information about the sVN branch were analyzed, the highest statistically significant agreement was found between the HvHIT and oVEMP tests (0.707; *p*< 0.05). In the IVN branch, only the PvHIT and cVEMP tests of the test methods we used were compared, and a statistically significant and moderate agreement was found. Finally, in the group with wVN, it is clearly seen that the use of vHIT and VEMP tests separately showed a statistically significant and good agreement. Unlike the studies mentioned above, we included oVEMP and cVEMP tests in the study and found that the VEMP test showed the most abnormal results in VN. Therefore, we suggest that the VEMP tests are needed to detect and differentiate types of VN.

Since there is a long and growing list of objective evaluations, a major question in vestibulogy is which test parameters agree with each other. We found that HvHIT and oVEMP are the most compatible superior nerve assessment tools. However, we suggested that no test should be used interchangeably. In contrast, to determine the type of VN, vHIT and VEMP should be used as a complete test battery. For inferior vestibular nerve assessment, a moderate relation was found between PvHIT and cVEMP. Evaluation of vertical canals has received less attention due to the associated technical difficulties. However, evaluation of the vertical canals provides deeper insights into vestibular disorders, especially for the disorders involving vertical canals, such as VN.

Regardless of inferior and superior nerve division, among all tests, HvHIT and PvHIT and oVEMP and cVEMP were found to be the tests that are the most in agreement. Although it is well known that the origin of cVEMP is from saccular and inferior vestibular nerve function and oVEMP predominantly originates from superior vestibular nerve function, there is an anterior (“hook”) region of the saccular macula that connects with superior vestibular neurons [18]. Therefore, the significant relationship between oVEMP and cVEMP is not. As a result, the strong link between oVEMP and cVEMP is not unexpected.

The current study has some limitations that should be acknowledged. Patients with all types of VN were included in the study. Had kappa analysis of CVT, HvHIT, and oVEMP tests been performed only for superior canal involvement, the results could have been more robust. The same limitation was valid for PvHIT, AvHIT, and cVEMP tests in patients with isolated inferior canal involvement of VN. Furthermore, the diagnosis of VN and determination of the area of involvement by MRI could be a more reliable analysis in terms of comparing the test consistency. However, since vestibular neuritis is the patient group with the highest prevalence after BPPV, applying the MRI method to this patient group is not seen as a cost-effective method in routine patient follow-up. As a result, evaluations were made in accordance with the clinical protocol.

The results of this study suggest that the most compatible tests are HvHIT and oVEMP for superior canal assessment and PvHITand cVEMP for inferior canal assessment. Regardless of nerve divisions, HvHIT and PvHIT are the objective tests in most agreement. This study will provide the backbone for evaluation of vestibular tests. Further studies should aim to include cutting-edge technologies, such as functional HIT [19] and ocular counter roll [20].

## Figures and Tables

**Table 1:** Demographics of patients.

Demographic features table		n	%	χ̄	SD
**Total**		37	100.00**%**	39.03	±11.67
**Sex**	*Famale*	19	51.35**%**	37.32	±13.30
	*Male*	18	48.65**%**	40.83	±9.72
**Age**	*18–30*	12	32.43**%**	25.50	±4.08
	*31–45*	13	35.14**%**	39.46	±4.77
	*46–60*	12	32.43**%**	52.08	±4.32

**n:** Number of the participants; χ̄: Mean; **SD**: Standard Deviation.

**Table 2:** Kappa values that indicate the degree of concordance between vestibular tests.

Branch of the vestibular nerve	Comparison tests Normal		Common results (%)			Cohen’s kappa coefficient (Κ)	
			Abnormal				
			Right	Left			
**Superior vestibular nerve**	CVT	HvHIT	37.80%	5.40%	10.80%	0.288*	Poor
	CVT	oVEMP	35.10%	5.40%	16.20%	0.262*	Poor
	CVT	AvHIT	26.60%	6.80%	13.60%	0.256*	Poor
	HvHIT	oVEMP	54.10%	8.10%	16.20%	0.707*	Good
	AvHIT	oVEMP	40.60%	16.20%	8.10%	0.585*	Moderate
**Inferior vestibular nerve**	PvHIT	cVEMP	27.10%	10.80%	16.20%	0.594*	Moderate
**Whole vestibular nerve**	oVEMP	cVEMP	18.92%	10.80%	24.30%	0.612*	Good
	HvHIT	PvHIT	35.20%	13.50%	13.50%	0.682*	Good

**CVT:** Caloric Vestibular Test, **vHIT:** Video Head Impulse Test, **HvHIT:** Horizontal vHIT, **AvHIT:** Anterior vHIT, **PvHIT**: Posterior vHIT, **oVEMP:** Ocular Vestibular Evoked Myogenic Potentials, **cVEMP**: Cervical Vestibular Evoked Myogenic Potentials.

* *p <* 0.05.
